# Risk factors and molecular features of sequence type (ST) 131 extended-Spectrum-β-lactamase-producing *Escherichia coli* in community-onset female genital tract infections

**DOI:** 10.1186/s12879-018-3168-8

**Published:** 2018-06-01

**Authors:** Young Ah Kim, Kyungwon Lee, Jae Eun Chung

**Affiliations:** 1 Department of Laboratory Medicine, National Health Insurance Service Ilsan Hospital, Baekseok-dong 1232, Ilsandong-gu, Goyang, 10444 South Korea; 2 Department of Laboratory Medicine, Yonsei University College of Medicine, 50-1 Yonsei-ro, Seodaemun-gu, Seoul 03722 South Korea; 3 Research Institute of Bacterial Resistance, Yonsei University College of Medicine, Seoul, South Korea; 4 Department of Obstetrics and Gynecology, National Health Insurance Service Ilsan Hospital, Baekseok-dong 1232, Ilsandong-gu, Goyang, 10444 South Korea

**Keywords:** Extended-spectrum beta-lactamase, *Escherichia coli*, Sequence type 131, Female genital tract infection

## Abstract

**Background:**

*Escherichia coli (E. coli)* is known to cause urinary tract infection (UTI) and meningitis in neonates, as well as existing as a commensal flora of the human gut. Extended-spectrum β-lactamase (ESBL)-producing *E. coli* has increased in the community with the spread of CTX-M type ESBL-producing sequence type 131 (ST131)-O25-*H30Rx E. coli* clone. The role of ESBL-producing *E. coli* in female genital tract infection has not been elucidated. The clinical and molecular features of *E. coli* isolated from community-onset female genital tract infections were evaluated to elucidate the current burden in the community, focusing on the highly virulent and multidrug-resistant ST131 clone.

**Methods:**

We collected and sequenced 91 non-duplicated *E. coli* isolates from the female genital tract of 514 patients with community-onset vaginitis. ESBL genotypes were identified by PCR and confirmed to be ESBL-producers by sequencing methods. ST131 clones were screened by PCR for O16-ST131 and O25b-ST131. Pulsed-field gel electrophoresis (PFGE) and PCR-based replicon typing (PBRT) were conducted in ESBL producers. Independent clinical risk factors associated with acquiring ESBL-producing *E. coli* and ST131 clone were analyzed using multivariate logistic regression analysis.

**Results:**

Of the 514 consecutive specimens obtained from the infected female genital tract, 17.7% (91/514) had *E. coli* infection, of which 19.8% (18/91) were ESBL producers. CTX-M-15 was the most common type (*n* = 15). O25b-ST131 and O16-ST131 clones accounted for 15.4% (14/91) and 6.6% (6/91), respectively. In plasmid analysis, ten isolates succeeded in conjugation and plasmid types were IncFII (*n* = 4), IncFI (*n* = 3), IncI1-Iγ (n = 3) with one non-typable case. Compared to ESBL-nonproducing *E. coli*, ESBL-producing *E. coli* acquisition was strongly associated with recurrent vaginitis (OR 40.130; 95% CI 9.980–161.366), UTI (OR 18.915; 95% CI 5.469–65.411), and antibiotics treatment (OR 68.390; 95% CI 14.870–314.531).

**Conclusion:**

A dominant clone of CTX-M type ESBL-producing *E. coli* in conjugative plasmids seems to be circulating in the community and considerable number of ST131 *E. coli* in the genital tract of Korean women was noted. Sustained monitoring of molecular epidemiology and control of the high-risk group is needed to prevent ESBL-producing *E. coli* from spreading throughout the community.

**Electronic supplementary material:**

The online version of this article (10.1186/s12879-018-3168-8) contains supplementary material, which is available to authorized users.

## Background

*Escherichia coli (E. coli)* is known to cause urinary tract infection (UTI), surgical site infection, and meningitis in neonates, as well as existing as a commensal flora of the human gut [1, 2]. The colonization of *E.coli* has been reported in both pregnant (24–31%) and non-pregnant (9–28%) women [3, 4]. The clinical risk factors for *E. coli* colonization or infection in the female genital tract is not fully understood, but postmenopausal changes including cessation of estrogen production, increased vaginal pH, disappearance of lactobacilli with the concurrent colonization of *Enterobacteriaceae* including *E. coli* is thought to play a role*.*^5^ Pregnant women with *E. coli* infection prompt additional attention due to its consequences regarding the pregnancy outcome including the infection of the new born [5].

In the past decade, colonization or infection of extended-spectrum-β-lactamase (ESBL)-producing *E. coli* has remarkably increased in the community, mostly due to the spread of high virulent and multidrug-resistant sequence type 131 (ST131) *E. coli* clone [6–8]. According to recent epidemiologic studies based on community-onset UTI, the situation of Korea is not different [6–8]. The presence of ESBL-producing *E. coli* in female genital tract infections are scarcely identified within our knowledge.

In this prospective observational study, risk factors and molecular features of female genital tract infections by *E. coli* were evaluated. We focused on the highly virulent and multidrug-resistant ST131 clone because it could be another indicator of the spread in the community and an emerging public health threat.

## Methods

For the prospective observational study, 91 non-duplicated *E. coli* isolates from the female genital tract specimens of 514 consecutive, sequentially encountered patients were analyzed. All specimen were retrieved from one community hospital in Gyeonggi-do province, South Korea (742 beds) under the diagnosis of community-onset vaginitis or cervicitis between June 2016 and April 2017. All patients had infection signs such as increased leucorrhoea, vaginal dyspareunia, intermittent pruritus, burning sensation, and foul odor. Sites of the acquisition were determined as described by Friedman with some modifications [9]. Community-onset was defined as diagnosis given within 48 h of admission, further categorized as community-onset healthcare-associated (COHA) and community-associated (CA) group. COHA infections had any one of the following histories: attended a hospital or hemodialysis clinic or received intravenous chemotherapy in the 30 days before the infection; hospitalized in an acute care hospital for 2 or more days in the 90 days, transfer-in from other healthcare facility before the infection. Others were defined as CA infection [9].

Species Identification and susceptibility testing were performed with Microscan Walk-away plus system (BeckmanCoulter, Inc., CA, USA) and MicroScan Neg Breakpoint Combo Type 44 (Siemens Healthcare Diagnostics Inc., West Sacramento, CA, USA). Antimicrobial susceptibility of the 91 *E. coli* isolates was tested and interpreted using CLSI criteria [10]. ESBL production was confirmed by ESBL double-disk synergy test [11], and ESBL genotype was determined by PCR and sequencing [12]. For the detection of ST131, all isolates were screened by PCR for O16-ST131, and O25b-ST131 [13]. Pulsed-field gel electrophoresis (PFGE) was performed as described in our previous study [14]. The patterns were analyzed using InfoQuest FP software (Bio-Rad) to generate a dendrogram based on the unweighted pair group method, with an arithmetic average (UPGMA) from the Dice coefficient with 1% band position tolerance and 0.5% optimization settings. A PCR based replicon typing (PBRT) were schemed in the 18 ESBL-producing *E. coli* isolates, targeting the replicons of the major plasmid families occurring in *Enterobacteriaceae* (HI2, HI1, I1-γ, X, L/M, N, FIA, FIB, FIC, W, Y, P, A/C, T, K, B/O) according to the protocol by Carattoli, et al. [15] Ten of the 18 isolates were successfully conjugated and PBRT was performed in these 10 transconjugants.

To find the independent clinical risk factors associated with acquiring the ESBL-producing *E. coli* and ST131 clone, medical records were reviewed for the patient’s age, pregnancy status, pregnancy outcome, menopause status, underlying medical conditions such as Diabetes Mellitus(DM) and hypertension, use of the intrauterine device or pessary, nursing home residency, pelvic inflammatory disease(PID), types of vaginitis such as atrophic vaginitis or chlamydial infection, previous antimicrobial treatment within 1 month, history of UTI within 1 month, and history of recurrent vaginitis within 1 month.

Statistical analysis was performed using Chi-square test for the comparative analysis of categorical variables to determine the independent risk factors. Fisher’s exact test was used when 25% of the cells had an expected frequency of less than 5. Odds ratio (OR) and 95% confidence interval (CI) values were calculated for binomial variables. Variables were first compared using univariate logistic regression, then a multivariate analysis using a backward selection with variables with a *p*-value < 0.1 in the univariate study was carried out. Multivariate logistic regression model was adjusted for age, pregnancy, intrauterine device use, menopause, admission from the nursing home, PID, recurrent vaginitis, UTI and previous antibiotic treatment within one month. Statistical significance was defined as *p* < 0.05. SPSS 17.0 (SPSS, Chicago, IL, USA) and SAS 9.4(SAS Institute Inc., Cary, NC) was used.

## Results

**Fig. 1:**
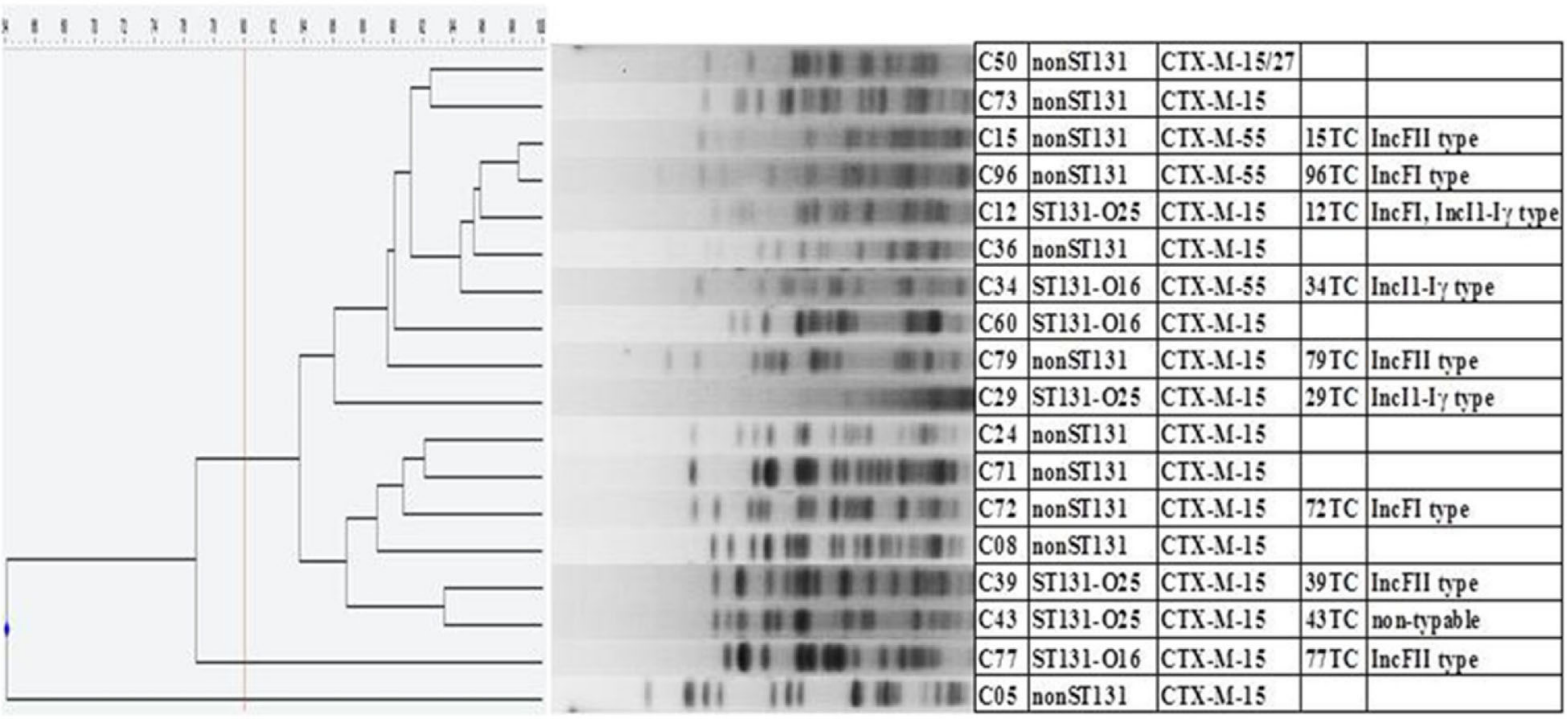
Pulsed-field gel electrophoresis of *χbal*-restricted DNA of community-onset ESBL-producing *Escherichia coli*, isolated from the infected female genital tract (*n* = 18)

Of the 514 consecutive specimens obtained from the infected female genital tract, 17.7% (91/514) had *E. coli* infection, of which 19.8% (18/91) were ESBL producers. All ESBL-producers had the CTX-M genotypes; CTX-M-15 was the most common type (*n* = 15), one of which also had CTX-M-27. CTX-M-55 producers were also detected (*n* = 3). Of the total 19 *E. coli* isolates, O25b-ST131 accounted for 15.4% (14/91) and O16-ST131 clones for 6.6% (6/91). Seven of the 18 ESBL producers were a ST131 clone with four being O25b-ST131 and three O16-ST131 (Table 1 and Additional file 1: Table S1). PFGE patterns of ESBL-producing *E. coli* showed three dominant clonal groups with the cut off 80% similarity, suggesting the clonal spread of ESBL-producing *E. coli* in the community (Fig. 1). In plasmid analysis, ten isolates succeeded in conjugation and plasmid types were IncFII (*n* = 4), IncFI (*n* = 3), IncI1-Iγ (n = 3) and one non-typable (Table 1). Antimicrobial susceptibility of the 91 *E. coli* isolates were analyzed. ST131 clone showed high resistance rates (RRs) to both 3rd generation cephalosporin and fluoroquinolones; 36% (O25b-ST131) versus 20% (non-ST131) to cefotaxime and 50% (O25b-ST131) versus 35% (non-ST131) to ciprofloxacin (Table 2 and Additional file 2: Table S2).

**Table 1:** Clinical and molecular features of community-onset ESBL-producing *Escherichia coli* isolated from the infected female genital tract (*n* = 18)

No	Age	Specimen	Accompanying disease	Site of acquisition	ESBL genotype	Plasmid type	ST131 (O25b/O16)
C05	39	Cervix	Preterm labor	CA	CTX-M − 15	–	Non ST131
C08	45	Cervix	Malaria	CA	CTX-M − 15	–	Non ST131
C12	96	Cervix	Cervix cancer, pneumonia	COHA	CTX-M − 15	IncFI, IncI1-Iγ,	ST131 (O25b)
C15	32	Cervix	Preterm labor	CA	CTX-M − 55	IncFII	Non ST131
C24	42	Cervix	Pelvic inflammatory disease	CA	CTX-M − 15	–	Non ST131
C29	54	Cervix	Atrophic vaginitis	CA	CTX-M − 15	IncI1-Iγ,	ST131 (O25b)
C34	31	Cervix	Preterm labor	CA	CTX-M − 55	IncI1-Iγ,	ST131 (O16b)
C36	51	Cervix	Recurrent vaginitis, UTI	CA	CTX-M − 15	–	Non ST131
C39	86	Cervix	Endometritis	COHA	CTX-M − 15	IncFII	ST131 (O25b)
C43	32	Cervix	Recurrent vaginitis, UTI	COHA	CTX-M − 15	non-typable	ST131 (O25b)
C50	34	Cervix	Pelvic inflammatory disease	CA	CTX-M − 15 CTX-M-27	–	Non ST131
C60	68	Cervix	Atrophic vaginitis	CA	CTX-M − 15	–	ST131 (O16)
C71	45	Cervix	Atrophic vaginitis	CA	CTX-M − 15	–	Non ST131
C72	46	Cervix	Recurrent vaginitis	CA	CTX-M-15	IncFI	Non ST131
C73	45	Cervix	Recurrent vaginitis	CA	CTX-M − 15	–	Non ST131
C77	32	Cervix	Pelvic inflammatory disease	CA	CTX-M − 15	IncFII	ST131 (O16)
C79	42	Cervix	Pelvic inflammatory disease	CA	CTX-M − 15	IncFII	Non ST131
C96	86	Endometrium	Atrophic vaginitis	COHA	CTX-M − 55	IncFI	Non ST131

*ESBL* Extended-spectrum-β-lactamase, *n* Number, *UTI* Urinary tract infection, *CA* Community-associated, *COHA* Community-onset, healthcare-associated; −, unconjugative plasmid, *ST* Sequence type, *O25b* Serogroup O25b, *O16* Serogroup O16

**Table 2:** Antimicrobial resistance rates (%) of *Escherichia coli* isolates of patients with female genital tract infection

Antimicrobial agent	Total (*n* = 91)	ESBL (*n* = 18)	Non-ESBL (*n* = 73)	ST131^a^ (*n* = 20)	O25b-ST131 (*n* = 14)	Non-ST131 (*n* = 71)
Ampicillin	67 (61/91)	100 (18/18)	59 (43/73)	85 (17/20)	79 (11/14)	62 (44/71)
Ampicillin/sulbactam	27 (25/91)	33 (6/18)	26 (19/73)	20 (4/20)	21 (3/14)	30 (21/71)
Piperacillin	65 (59/91)	100 (18/18)	56 (41/73)	85 (17/20)	79 (11/14)	59 (42/71)
Piperacillin/Tazobactam	0 (0/91)	0 (0/18)	0 (0/73)	0 (0/20)	0 (0/14)	0 (0/71)
Cefuroxime	25 (23/91)	94 (17/18)	8 (6/73)	40 (8/20)	36 (5/14)	21 (15/71)
Cefotaxime	25 (23/91)	94 (17/18)	7 (5/73)	40 (8/20)	36 (5/14)	20 (14/71)
Ceftazidime	24 (22/91)	94 (17/18)	7 (5/73)	40 (8/20)	36(5/14)	20 (14/71)
Cefoxitin	7 (6/91)	6 (1/18)	7 (5/73)	5 (1/20)	7 (1/14)	7 (5/71)
Cefepime	22 (20/91)	100 (18/18)	3 (2/73)	35 (7/20)	29 (4/14)	18 (13/71)
Aztreonam	22 (20/91)	94 (17/18)	4 (3/73)	35 (7/20)	29 (4/14)	18 (13/71)
Meropenem	0 (0/91)	0 (0/18)	0 (0/73)	0 (0/20)	0 (0/14)	0 (0/71)
Doripenem	0 (0/91)	0 (0/18)	0 (0/73)	0 (0/20)	0 (0/14)	0 (0/71)
Imipenem	0 (0/91)	0 (0/18)	0 (0/73)	0 (0/20)	0 (0/14)	0 (0/71)
Ciprofloxacin	36 (33/91)	67 (12/18)	29 (21/73)	40 (8/20)	50 (7/14)	35 (25/71)
Levofloxacin	30 (27/91)	61 (11/18)	22 (16/73)	40 (8/20)	50 (7/14)	27 (19/71)
Gentamicin	25 (23/91)	33 (6/18)	23 (17/73)	25 (5/20)	21 (3/14)	25 (18/71)
Tobramycin	18 (16/91)	22 (4/18)	16 (12/73)	15 (3/20)	14 (2/14)	18 (13/71)
Amikacin	1 (1/91)	0 (0/18)	1 (1/73)	0 (0/20)	0 (0/14)	1 (1/71)
Cotrimoxazole	40 (36/91)	44 (8/18)	38 (28/73)	45 (9/20)	43 (6/14)	38 (27/71)
Tigecycline	0 (0/91)	0 (0/18)	0 (0/73)	0 (0/20)	0 (0/14)	0 (0/71)
Colistin	0 (0/91)	0 (0/18)	0 (0/73)	0 (0/20)	0 (0/14)	0 (0/71)

^a^*ST131* Sequence type131 (both O25 and O16), *O25b* Serogroup O25b, *O16* Serogroup O16, ESBL, ESBL-producer; non-ESBL; non-ESBL producer

The median age of the patients with ESBL-producing *E. coli* was 45 years old (31–96) and accompanying diseases were recurrent vaginitis (*n* = 4), atrophic vaginitis (*n* = 3), pelvic inflammatory disease (*n* = 3), preterm labor (*n* = 3), and UTI (*n* = 2). They were treated with a variety of antimicrobial therapies, which included cephalosporin, carbapenems, piperacillin-tazobactam, and amikacin, alone or in combination and achieved a clinical cure. All three pregnancies with ESBL-producing *E. coli* female genital tract infection were prematurely terminated due to preterm labor (Table 1).

Independent clinical risk factors for acquiring the ESBL-producing *E. coli* over non-ESBL producing *E. coli* were preterm labor (OR, 10.911, 95% CI, 1.199–99.301; *P* = 0.0339), intrauterine device insertion (OR, 6.460; 95% CI, 1.004–41.568; *P* = 0.0495), history of recurrent vaginitis within one month (OR, 40.130; 95% CI, 9.980–161.366; *P* ≤ 0.0001), history of UTI within one month (OR, 18.915; 95% CI, 5.469–65.411; *P* ≤ 0.0001), and antibiotics treatment within one month (OR, 68.390; 95% CI, 14.870–314.531; *P* ≤ 0.0001) (Table 3) .

**Table 3:** Clinical risk factors of acquiring the ESBL-producing *Escherichia coli* and ST131 clone

Risk factors	ESBL vs non-ESBL				ST131 vs non-ST131			
	N (%)	OR	95% CI	*P*	N (%)	OR	95% CI	*P*
Preterm labor in pregnancy	3(16.7)	10.911	1.199–99.301	0.0339	–	–	–	–
Age								
30–39	6(33.3)				5(25.0)			
40–49	6(33.3)	1.057	0.289–3.861	0.9330	3(15.0)	0.828	0.170–4.026	0.5497
50–59	2(11.1)	0.385	0.075–1.967	0.2512	4(20.0)	5.541	0.130–236.943	0.8151
> = 60	4(22.2)	0.625	0.156–2.494	0.5054	8(40.0)	5.130	0.099–266.909	0.3716
Menopause	6(33.3)	0.0365	0.126–1.059	0.0636	12(60.0)	0.208	0.005–8.843	0.4118
IUD	3(16.7)	6.460	1.004–41.568	0.0495	1(5.0)	0.965	0.088–10.645	0.9770
Recurrent within 1 month	14(77.8)	40.130	9.980–161.366	< 0.0001	6(30.0)	1.883	0.105–33.820	0.6677
Nursing home T/I	3(16.7)	3.488	0.709–17.152	0.1242	3(15.0)	1.088	0.096–12.292	0.9459
PID	14(77.8)	0.489	0.113–2.122	0.3396	10(50.0)	0.744	0.164–3.378	0.7017
UTI^a^	9(50.0)	18.915	5.469–65.411	< 0.0001	11(55.0)	1.513	0.362–6.319	0.5506
Previous antibiotics^a^	15(83.3)	68.390	14.870–314.531	< 0.0001	6(30.0)	1.172	0.063–21.644	0.9149

ESBL, female genital tract infections with ESBL-producing *E. coli*; Non-ESBL, female genital tract infections with non-ESBL-producing *E. coli*; OR, odds ratio; 95% CI, 95% confidential interval; T/I, transfer-in; IUD, intrauterine device; PID, pelvic inflammatory disease; UTI, urinary tract infection; ^a^ within one month

Multivariate analysis adjusted for age, pregnancy, intrauterine device use, menopause, admission from the nursing home, PID, recurrent vaginitis, UTI and previous antibiotic treatment within one month

Clinical characteristics were compared between the ST131 and non-ST131 clone. In the multivariate logistic regression analysis adjusting for the compounding variables, independent risk factors with statistical significance could not be found that were associated with acquiring the ST131 clone (Table 3).

## Discussion

ESBL-producing *E. coli* was considered to be a crucial nosocomial pathogen when it was first isolated in the late 1980s [6–8]. With the occurrence of the ST131 clone, ESBL-producing *E. coli* in the community setting has been widely noted in recent studies [16–18]. Our previous study showed that 27% (58/213) of *E. coli* isolates from UTI patients belonged to the globally epidemic ST131 clone [6]. The Korean Antimicrobial Resistance Monitoring System (KARMS) reported the resistance rates of *E. coli* to cefotaxime to be 35% in 2015 [19]. Asymptomatic carriage of ESBL-producing *E. coli* among healthy individuals playing the role of reservoir are also known to have increased [20]. Fecal colonization with ESBL-producing *E. coli* are also on the rise [21].

Analysis of vaginal microbiome showed region-specific variation, setting the basis of the need for a region-specific data of the epidemic multi-drug resistant ST131 ESBL-producing *E. coli* clone [22, 23]. We evaluated the prevalence of ESBL-producing *E. coli,* focusing on the ST131 clone in female genital tract infections because it could be another indicator of the spread of highly virulent and multi-drug resistant *E. coli* in the community, which could be an emerging threat to the public health.

Of the 514 consecutive specimens obtained from the infected female genital tract, 17.7% (91/514) had *E. coli* infection, of which 19.8% (18/91) were ESBL producers. Fourteen of the 18 ESBL producers were community-associated(CA) without any history of hospitalization, suggesting the possibility of community based spread of ESBL-producing *E. coli* (Table 1).

CTX-M-15 was the most common type (*n* = 15) and this was in accordance of the previous isolates of CTX types in Korea in which either CTX-M-1 group (including CTX-M-15 or CTX-M-55) or CTX-M-9 group (including CTX-M-14 or CTX-M-27) prevailed [6, 7]. CTX-M-55 genotype is a variant of CTX-M-15 with a single amino acid substitution, which was known to frequent in China and considered as a potential threat to community spread [24]. Recently CTX-M-55-producing *Shigella* and *Salmonella* were isolated in Korea with *bla*_CTX-M-55_ genes inserted into IncI1, IncA/C, and IncZ plasmid, downstream of IS*Ecp1*, IS*26-*IS*Ecp1* and IS*Ecp-*IS*5* sequences, which suggests CTX-M-55 dissemination to different bacterial species by lateral plasmid transfer [25]. In this study, ten of 18 ESBL-producing *E. coli* succeeded in conjugation, and plasmid types were IncFII (*n* = 4), IncFI (*n* = 3), IncI1-Iγ (n = 3) with one non-typable case. Most of ESBL-producing *E. coli* showed similar clonality in PFGE, which suggests that dominant clone of CTX-M type ESBL-producing *E. coli* in conjugative plasmids are circulating in the community.

O25b-ST131 and O16-ST131 clones accounted for 15.4% (14/91) and 6.6% (6/91), respectively, suggesting a high prevalence of ST131 *E. coli* clone in the female genital tracts of Korean women. The expansion of the ST131 *E. coli* clone with phylogenetic B2, serotype O25b, *fimH* type *H30* is suggested to reveal multidrug resistant property [8, 17]. In this study, O25b-ST131 clones showed high rates of multidrug-resistancy against the 3rd generation cephalosporins as well as fluoroquinolones (Table 2 and Additional file 2: Table S2).

Compared to the ESBL-nonproducing *E. coli* group, ESBL-producing *E. coli* group showed higher tendency of having clinical features such as history of recurrent vaginitis within 1 month (OR, 40.130; 95% CI, 9.980–161.366; *P* ≤ 0.0001), history of UTI within 1 month (OR, 18.915; 95% CI, 5.469–65.411; *P* ≤ 0.0001), and history or antibiotics treatment within 1 month (OR, 68.390; 95% CI, 14.870–314.531; *P* ≤ 0.0001). *E. coli* is a well-known etiologic agent for UTI, so it is not surprising that female genital tract infection with ESBL-producing *E. coli* were strongly associated with UTI [6–8]. Previous antibiotic exposure might have resulted in the selection pressure of a resistant clone, concomitantly developing multidrug-resistancy [14, 26].

Aerobic vaginitis is a recently defined vaginitis type, which differs from the common bacterial vaginosis in terms of scarce presence of lactobacilli and positive cultured aerobic bacteria including group B streptococci, *E.coli*, and enterococci [5]. In postmenopausal women with atrophic vaginitis, the lack of estrogen results in deficiency of mucosal epithelial barrier, lactobacilli disappear from the vaginal flora decreasing the pH of the vagina, resulting in predominant colonization by *Enterobacteriaceae*, especially *E. coli.*, serving an adequate environment for aerobic vaginitis to set place [2, 27].

In cases of pregnancy, aerobic vaginitis is known to be associated with an increased risk of preterm labor and chorioamnionitis [5]. Although all three pregnancies infected by ESBL producers resulted in preterm labor in this study, due to the small number of included patients and the omission of cases without genital tract infection, the role of ESBL producers in poor pregnancy outcome should be interpreted with caution. Further studies including a larger number of pregnancies to elucidate the role of ESBL-producing *E. coli* in the genital tract infection is warranted.

## Conclusions

In conclusion, a dominant clone of CTX-M type ESBL-producing *E. coli* in conjugative plasmids seems to be circulating in the community and considerable number of ST131 *E. coli* clone in the genital tracts of Korean women was noted. Sustained monitoring of molecular epidemiology and an adequate control of the clinically high-risk group is needed to prevent ESBL-producing *E. coli* from spreading throughout the community.

## Additional files

Additional file 1: **Table S1.** Primer sequences for ESBL genotyping used in this study. Target genes, primer name, and primer sequences are shown. (DOCX 16 kb)

Additional file 2: **Table S2.** Genetic information and antimicrobial susceptibility of *Escherichia coli* from female genital tract. Detailed information concerning the 91 *E.coli* specimens is included. (DOCX 40 kb)
