# Supplementary material for: Risk factors and molecular features of sequence type (ST) 131 extended-Spectrum-β-lactamase-producing Escherichia coli in community-onset female genital tract infections
Source: BMC Infect Dis. 2018 Jun 1;18:250. doi: 10.1186/s12879-018-3168-8 (PMC5984740; doi:10.1186/s12879-018-3168-8)
Supplement: Supplementary file 1 — Table S1. Primer sequences for ESBL genotyping used in this study. Target genes, primer name, and primer sequences are shown. (DOCX 16 kb) [file 12879_2018_3168_MOESM1_ESM.docx]

| Table S1. Primer sequences for ESBL genotyping used in this study^13^ | | | | |
| --- | --- | --- | --- | --- |
| Target genes | Primer name | Sequence (5'→3') | Annealing  temperature | Product  size (bp) |
| *bla*_TEM_ | TEM F | ATGAGTATTCAACATTTCCGT | 59℃ | 861 |
|  | TEM R | TTACCAATGCTTAATCAGTGA |  |  |
| *bla*_SHV_ | SHV F | CCGGGTTATTCTTATTTGTCGCT | 61℃ | 927 |
|  | SHV R | TAGCGTTGCCAGTGCTCG |  |  |
| *bla*_CTX-M_ | CTXM-1 F | ACCGTCACGCTGTTGTTAGG | 56℃ | 819 |
|  | CTXM-1 R | CAAGGTGACGATTTTAGCCG |  |  |
|  | CTXM-2 F | AATGTTAACGGTGATGGCGA | 56℃ | 844 |
|  | CTXM-2 R | ACCGTGGGTTACGATTTTCG |  |  |
|  | CTXM-9 F | GTGCAACGGATGATGTTCG | 56℃ | 845 |
|  | CTXM-9 R | ATGATTCTCGCCGCTGAAG |  |  |
|  | CTXM-25 F | GTAAGGCGGGCGATGTTAAT | 56℃ | 856 |
|  | CTXM-25 R | AACCGTCGGTGACAATTCTG |  |  |
| ESBL, extended-spectrum-β-lactamase; bp, base pair | | | | |
