# Supplementary material for: Risk factors and molecular features of sequence type (ST) 131 extended-Spectrum-β-lactamase-producing Escherichia coli in community-onset female genital tract infections
Source: BMC Infect Dis. 2018 Jun 1;18:250. doi: 10.1186/s12879-018-3168-8 (PMC5984740; doi:10.1186/s12879-018-3168-8)
Supplement: Supplementary file 2 — Table S2. Genetic information and antimicrobial susceptibility of Escherichia coli from female genital tract. Detailed information concerning the 91 E.coli specimens is included. (DOCX 40 kb) [file 12879_2018_3168_MOESM2_ESM.docx]

Table S2. Genetic information and antimicrobial susceptibility of *Escherichia coli* from female genital tract.

| No | Specimen | ESBL-genotype | ST131 PCR | Plasmid type | A  M  K | AMP | S  AM | A  TM | FEP | CTX | FOX | CAZ | CXM | C  I  P | COL | SXT | DOR | GEN | IMP | LEV | MEM | P  I  P | TZP | TGC | TOB |
| --- | --- | --- | --- | --- | --- | --- | --- | --- | --- | --- | --- | --- | --- | --- | --- | --- | --- | --- | --- | --- | --- | --- | --- | --- | --- |
| C002 | Cervix |  | O25b-ST131 |  | S | R | R | S | S | R | R | R | R | S | S | R | S | S | S | S | S | R | S | S | S |
| C004 | Cervix |  | O16-ST131 |  | S | R | I | S | S | S | S | S | S | S | S | R | S | R | S | S | S | R | S | S | R |
| C005 | Cervix | CTX-M-15 | negative | unconjugative | S | R | R | R | R | R | S | R | R | R | S | R | S | S | S | I | S | R | S | S | S |
| C007 | Cervix |  | negative |  | S | R | I | S | S | S | S | S | S | S | S | S | S | S | S | S | S | R | S | S | S |
| C008 | Cervix | CTX-M-15 | negative | unconjugative | S | R | I | R | R | R | S | R | R | R | S | S | S | S | S | R | S | R | S | S | S |
| C009 | Cervix |  | O25b-ST131 |  | S | R | I | S | S | S | S | S | S | S | S | R | S | S | S | S | S | R | S | S | S |
| C010 | Cervix |  | O25b-ST131 |  | S | S | S | S | S | S | S | S | S | S | S | S | S | S | S | S | S | S | S | S | S |
| C012 | Cervix | CTX-M-15 | O25b-ST131 | IncFI, IncI1-Iγ, | S | R | I | R | R | R | S | R | R | R | S | S | S | R | S | R | S | R | S | S | I |
| C013 | Cervix |  | negative |  | S | R | R | S | S | S | S | S | S | R | S | S | S | S | S | R | S | R | S | S | S |
| C014 | Cervix |  | negative |  | S | S | S | S | S | S | S | S | S | S | S | S | S | S | S | S | S | S | S | S | S |
| C015 | Cervix | CTX-M-55 | negative | IncFII | S | R | I | R | R | R | S | R | R | S | S | R | S | R | S | S | S | R | S | S | I |
| C016 | Cervix |  | negative |  | S | S | S | S | S | S | S | S | S | S | S | S | S | S | S | S | S | S | S | S | S |
| C017 | Cervix |  | negative |  | S | S | S | S | S | S | I | S | S | S | S | S | S | S | S | S | S | S | S | S | S |
| C018 | Cervix |  | negative |  | S | S | S | S | S | S | S | S | S | S | S | S | S | S | S | S | S | S | S | S | S |
| C019 | Cervix |  | negative |  | S | R | I | S | S | S | S | S | S | R | S | S | S | R | S | R | S | R | S | S | R |
| C020 | Cervix |  | negative |  | S | R | R | S | S | S | S | S | S | R | S | R | S | R | S | R | S | R | S | S | R |
| C021 | Cervix |  | negative |  | S | R | I | I | S | S | S | S | S | S | S | R | S | S | S | S | S | R | S | S | S |
| C022 | Vagina |  | negative |  | S | R | R | R | R | R | R | R | R | R | S | R | S | S | S | I | S | R | S | S | S |
| C023 | Vagina |  | O25b-ST131 |  | S | S | S | S | S | S | S | S | S | S | S | S | S | S | S | S | S | S | S | S | S |
| C024 | Cervix | CTX-M-15 | negative | unconjugative | I | R | R | R | R | R | S | R | R | R | S | S | S | S | S | R | S | R | S | S | R |
| C025 | Cervix |  | negative |  | S | R | I | S | S | S | S | S | S | R | S | R | S | S | S | I | S | R | S | S | S |
| C026 | Cervix |  | negative |  | S | R | S | R | S | S | S | S | S | S | S | R | S | S | S | S | S | R | S | S | S |
| C027 | Cervix |  | negative |  | S | S | S | S | S | S | S | S | S | S | S | S | S | S | S | S | S | S | S | S | S |
| C028 | Cervix |  | negative |  | S | R | R | S | S | S | S | S | S | S | S | R | S | R | S | S | S | R | S | S | R |
| C029 | Cervix | CTX-M-15 | O25b-ST131 | IncI1-Iγ, | S | R | R | R | R | R | S | R | R | R | S | R | S | S | S | R | S | R | S | S | S |
| C030 | Cervix |  | negative |  | S | R | R | S | S | S | S | S | S | S | S | R | S | R | S | S | S | R | S | S | I |
| C031 | Cervix |  | negative |  | S | S | S | S | S | S | S | S | S | S | S | R | S | R | S | S | S | S | S | S | R |
| C032 | Cervix |  | negative |  | S | S | S | S | S | S | S | S | S | S | S | S | S | S | S | S | S | S | S | S | S |
| C033 | Vagina |  | negative |  | S | R | R | I | S | R | R | R | R | S | S | R | S | S | S | S | S | R | S | S | S |
| C034 | Cervix | CTX-M-55 | O16-ST131 | IncI1-Iγ, | S | R | I | R | R | R | S | R | R | S | S | S | S | S | S | S | S | R | S | S | S |
| C035 | Cervix |  | negative |  | S | R | R | S | S | S | S | S | S | S | S | R | S | S | S | S | S | R | S | S | S |
| C036 | Cervix | CTX-M-15 | negative | unconjugative | S | R | R | R | R | R | S | R | R | R | S | S | S | S | S | R | S | R | S | S | S |
| C037 | Cervix |  | negative |  | S | R | R | S | S | S | S | S | S | S | S | R | S | R | S | S | S | R | S | S | R |
| C038 | Cervix |  | negative |  | S | R | I | S | S | S | S | S | S | S | S | S | S | S | S | S | S | R | S | S | S |
| C039 | Endometrium | CTX-M-15 | O25b-ST131 | IncFII | S | R | I | R | R | R | S | R | R | R | S | S | S | R | S | R | S | R | S | S | R |
| C041 | Vagina |  | negative |  | S | S | S | S | S | S | S | S | S | S | S | S | S | S | S | S | S | S | S | S | S |
| C042 | Cervix |  | negative |  | S | S | S | S | S | S | S | S | S | S | S | S | S | S | S | S | S | S | S | S | S |
| C043 | Cervix | CTX-M-15 | O25b-ST131 | non-typable | S | R | I | R | R | R | S | R | R | R | S | R | S | R | S | R | S | R | S | S | R |
| C044 | Cervix |  | negative |  | S | R | I | S | S | S | S | S | S | R | S | R | S | R | S | R | S | R | S | S | I |
| C045 | Cervix |  | negative |  | S | R | R | R | R | R | R | R | R | R | S | S | S | R | S | I | S | R | S | S | R |
| C046 | Cervix |  | negative |  | S | S | S | S | S | S | S | S | S | S | S | S | S | S | S | S | S | S | S | S | S |
| C047 | Cervix |  | negative |  | S | R | I | S | S | S | I | S | R | S | S | S | S | S | S | S | S | R | S | S | S |
| C048 | Cervix |  | negative |  | S | S | S | S | S | S | S | S | S | S | S | S | S | S | S | S | S | S | S | S | S |
| C049 | Cervix |  | O25b-ST131 |  | S | R | I | S | S | S | S | S | S | R | S | S | S | S | S | R | S | R | S | S | S |
| C050 | Cervix | CTX-M-15, CTX-M-27 | negative | unconjugative | S | R | S | R | R | R | S | R | R | R | S | R | S | S | S | R | S | R | S | S | S |
| C051 | Cervix |  | negative |  | S | S | S | S | S | S | S | S | S | S | S | S | S | S | S | S | S | S | S | S | S |
| C052 | Cervix |  | negative |  | S | S | S | S | S | S | S | S | S | R | S | S | S | S | S | R | S | S | S | S | S |
| C053 | Cervix |  | negative |  | S | R | I | S | S | S | S | S | S | S | S | S | S | S | S | S | S | R | S | S | S |
| C054 | Cervix |  | O25b-ST131 |  | S | S | S | S | S | S | S | S | S | S | S | S | S | S | S | S | S | S | S | S | S |
| C055 | Cervix |  | negative |  | S | S | S | S | S | S | S | S | S | R | S | S | S | S | S | R | S | S | S | S | S |
| C056 | Cervix |  | negative |  | S | R | S | S | S | S | S | S | S | R | S | R | S | R | S | I | S | R | S | S | I |
| C057 | Cervix |  | negative |  | S | S | S | S | S | S | S | S | S | S | S | S | S | S | S | S | S | S | S | S | S |
| C058 | Cervix |  | negative |  | S | R | R | S | S | S | S | S | S | R | S | R | S | R | S | R | S | R | S | S | R |
| C059 | Cervix |  | negative |  | S | S | S | S | S | S | S | S | S | S | S | S | S | S | S | S | S | S | S | S | S |
| C060 | Cervix | CTX-M-15 | O16-ST131 | unconjugative | S | R | I | R | R | R | S | R | R | S | S | R | S | S | S | S | S | R | S | S | S |
| C062 | Cervix |  | negative |  | S | R | I | S | S | S | S | S | S | S | S | S | S | S | S | S | S | R | S | S | S |
| C063 | Cervix |  | negative |  | S | R | S | S | S | S | S | S | S | R | S | S | S | R | S | R | S | R | S | S | R |
| C064 | Cervix |  | negative |  | S | R | I | S | S | S | S | S | S | S | S | R | S | S | S | S | S | R | S | S | S |
| C065 | Cervix |  | negative |  | S | S | S | S | S | S | S | S | S | S | S | S | S | S | S | S | S | S | S | S | S |
| C066 | Cervix |  | negative |  | S | R | I | S | S | S | S | S | S | S | S | R | S | S | S | S | S | R | S | S | I |
| C067 | Cervix |  | negative |  | S | R | R | I | S | R | R | R | R | S | S | S | S | S | S | S | S | I | S | S | S |
| C068 | Cervix |  | negative |  | S | R | I | S | S | R | I | S | I | S | S | R | S | R | S | S | S | R | S | S | I |
| C069 | Cervix |  | negative |  | S | S | S | S | S | S | S | S | S | S | S | S | S | S | S | S | S | S | S | S | S |
| C070 | Cervix |  | negative |  | S | R | S | S | S | S | S | S | S | R | S | R | S | S | S | I | S | R | S | S | S |
| C071 | Cervix | CTX-M-15 | negative | unconjugative | S | R | R | R | R | R | R | R | R | R | S | S | S | S | S | R | S | R | S | S | S |
| C072 | Cervix | CTX-M-15 | negative | IncFI | S | R | R | R | R | R | I | R | R | R | S | R | S | R | S | R | S | R | S | S | R |
| C073 | Cervix | CTX-M-15 | negative | unconjugative | S | R | I | R | R | R | S | R | R | S | S | R | S | S | S | S | S | R | S | S | S |
| C074 | Cervix |  | negative |  | S | S | S | S | S | S | S | S | S | S | S | S | S | S | S | S | S | S | S | S | S |
| C075 | Cervix |  | negative |  | S | S | S | S | S | S | S | S | S | S | S | S | S | S | S | S | S | S | S | S | S |
| C076 | Cervix |  | negative |  | S | R | R | S | S | S | S | S | S | S | S | R | S | R | S | S | S | R | S | S | I |
| C077 | Cervix | CTX-M-15 | O16-ST131 | IncFII | S | R | I | R | R | R | S | R | R | S | S | S | S | R | S | S | S | R | S | S | I |
| C078 | Cervix |  | negative |  | S | S | S | S | S | S | S | S | S | R | S | S | S | S | S | R | S | S | S | S | S |
| C079 | Cervix | CTX-M-15 | negative | IncFII | S | R | I | S | S | S | S | S | S | S | S | S | S | S | S | S | S | R | S | S | S |
| C080 | Cervix |  | negative |  | S | S | S | S | S | S | S | S | S | S | S | S | S | S | S | S | S | S | S | S | S |
| C082 | Cervix |  | negative |  | R | R | R | S | S | S | S | S | S | R | S | R | S | R | S | R | S | I | S | S | R |
| C083 | Cervix |  | negative |  | S | R | R | S | S | S | S | S | S | R | S | R | S | S | S | R | S | R | S | S | S |
| C084 | Cervix |  | O25b-ST131 |  | S | R | R | S | S | S | S | S | S | R | S | R | S | S | S | R | S | R | S | S | S |
| C085 | Cervix |  | negative |  | S | S | S | S | S | S | S | S | S | S | S | S | S | S | S | S | S | S | S | S | S |
| C086 | Vagina |  | O16-ST131 |  | S | R | R | S | S | S | S | S | S | R | S | S | S | S | S | R | S | R | S | S | S |
| C087 | Vagina |  | O25b-ST131 |  | S | R | I | S | S | S | S | S | S | R | S | S | S | S | S | R | S | R | S | S | S |
| C088 | Cervix |  | negative |  | S | S | S | S | S | S | S | S | S | S | S | S | S | S | S | S | S | S | S | S | S |
| C090 | Cervix |  | negative |  | S | S | S | S | S | S | S | S | S | S | S | S | S | S | S | S | S | S | S | S | S |
| C091 | Cervix |  | O16-ST131 |  | S | R | I | S | S | S | S | S | S | S | S | R | S | S | S | S | S | R | S | S | S |
| C092 | Cervix |  | negative |  | S | R | R | S | S | S | S | S | S | S | S | S | S | S | S | S | S | R | S | S | S |
| C093 | Cervix |  | negative |  | S | S | S | S | S | S | S | S | S | S | S | S | S | S | S | S | S | S | S | S | S |
| C094 | Cervix |  | negative |  | S | R | R | S | S | S | S | S | S | S | S | R | S | R | S | S | S | R | S | S | R |
| C096 | Cervix | CTX-M-55 | negative | IncFI | S | R | S | R | R | R | S | R | R | R | S | S | S | S | S | R | S | R | S | S | S |
| C097 | Cervix |  | negative |  | S | R | I | S | S | S | S | S | S | R | S | S | S | R | S | R | S | R | S | S | R |
| C098 | Cervix |  | O25b-ST131 |  | S | R | I | S | S | S | S | S | S | S | S | S | S | S | S | S | S | R | S | S | S |
| C099 | Cervix |  | O25b-ST131 |  | S | R | S | S | S | S | S | S | S | S | S | R | S | S | S | S | S | R | S | S | S |
| C100 | Cervix |  | negative |  | S | S | S | S | S | S | S | S | S | S | S | S | S | S | S | S | S | S | S | S | S |

ESBL, extended-spectrum-β-lactamase; AMK, Amikacin; AMP, Ampicillin; SAM, Ampicillin/sulbactam; ATM, Aztreonam; FEP, Cefepime ; CTX, Cefotaxime; FOX, Cefoxitin ; CAZ, Ceftazidime; CXM, Cefuroxime; CIP; Ciprofloxacin; COL, Colistin; SXT, Cotrimoxazole; DOR, Doripenem; GEN, Gentamicin; IMP, Imipenem; LEV, Levofloxacin; MEM, Meropenem; PIP, Piperacillin; TZP, Piperacillin/Tazobactam; TGC, Tigecycline; TOB, Tobramycin
